# *ADCY5*-related dyskinesia presenting as familial myoclonus-dystonia

**DOI:** 10.1007/s10048-017-0510-z

**Published:** 2017-02-22

**Authors:** Andrew G. L. Douglas, Gaia Andreoletti, Kevin Talbot, Simon R. Hammans, Jaspal Singh, Andrea Whitney, Sarah Ennis, Nicola C. Foulds

**Affiliations:** 1 Academic Unit of Human Development and Health, Faculty of Medicine, University of Southampton, Southampton, UK; 2 Wessex Clinical Genetics Service, University Hospital Southampton NHS Foundation Trust, Level G, Mailpoint 627, Princess Anne Hospital, Coxford Road, Southampton, SO16 5YA UK; 3 Nuffield Department of Clinical Neurosciences, University of Oxford, Oxford, UK; 4 Wessex Neurological Centre, University Hospital Southampton NHS Foundation Trust, Southampton, UK; 5 Paediatric Neurology, University Hospital Southampton NHS Foundation Trust, Southampton, UK

**Keywords:** ADCY5, Familial dyskinesia, Myoclonus-dystonia, Exome sequencing

## Abstract

We describe a family with an autosomal dominant familial dyskinesia resembling myoclonus-dystonia associated with a novel missense mutation in *ADCY5*, found through whole-exome sequencing. A tiered analytical approach was used to analyse whole-exome sequencing data from an affected grandmother-granddaughter pair. Whole-exome sequencing identified 18,000 shared variants, of which 46 were non-synonymous changes not present in a local cohort of control exomes (*n* = 422). Further filtering based on predicted splicing effect, minor allele frequency in the 1000 Genomes Project and on phylogenetic conservation yielded 13 candidate variants, of which the heterozygous missense mutation c.3086T>G, p. M1029R in *ADCY5* most closely matched the observed phenotype. This report illustrates the utility of whole-exome sequencing in cases of undiagnosed movement disorders with clear autosomal dominant inheritance. Moreover, *ADCY5* mutations should be considered in cases with apparent myoclonus-dystonia, particularly where *SCGE* mutations have been excluded. *ADCY5*-related dyskinesia may manifest variable expressivity within a single family, and affected individuals may be initially diagnosed with differing neurological phenotypes.

**Electronic supplementary material:**

The online version of this article (doi:10.1007/s10048-017-0510-z) contains supplementary material, which is available to authorized users.

## Introduction

Heterozygous *ADCY5* mutations, encoding adenylyl cyclase 5, cause familial dyskinesia with facial myokymia (FDFM; OMIM:606703) and benign hereditary chorea (BHC; OMIM:118700), comprising ‘*ADCY5*-related dyskinesia’ [1–5]. We report a three-generation family with *ADCY5*-related dyskinesia manifesting as myoclonus-dystonia, caused by a novel missense variant identified through whole-exome sequencing (WES) of a grandmother-granddaughter pair.

## Case summaries

### Proband (IV:1)

A 13-year-old female presented aged 9 years with a dyskinetic movement disorder. Early development was normal, but at 2 years, she developed unsteady gait with dystonic lower limb posturing and upper limb jerks, frequently dropping objects when tired. General health was good with no learning difficulties. Her condition was initially paroxysmal but deteriorated over several years, becoming a persistent, dystonic, myoclonic movement disorder. Facial jerking and oro-lingual dystonia were particularly noticeable and functionally impairing, affecting speech and causing drooling. Dystonia predominantly affected her upper limbs with minimal trunk involvement. Saccadic eye movements were jerky with intermittent overshooting; visual acuity was normal. There was no trunk or limb ataxia. She remained ambulant but had significant functional fine motor difficulties. Symptoms worsened with anxiety or concentration. Clonazepam worsened symptoms and negatively affected mood. Levitiracetam and trihexyphenidyl were unbeneficial. Array comparative genomic hybridisation, *SGCE* and *NKX2-1* Sanger sequencing were normal, as were brain MRI and neurometabolic investigations. Cerebrospinal fluid 5-hydroxyindoleacetic acid was marginally low [43 nmol/L (ref: 58–220)], reflecting impaired serotonin turnover.

### Proband’s mother (III:4)

This 32-year-old woman developed involuntary movements aged 2 years. Her symptoms progressed, particularly in her twenties. Movements predominantly affected upper limbs, head and neck, being less apparent in lower limbs. Symptoms worsened with tiredness and emotional stress. She had choreiform movements, a ‘no-no’ head tremor and marked dystonia of shoulders and upper limbs, especially on movement. Eye movements were normal with intact finger-nose pointing and no dysdiadochokinesis. However, heel-toe tandem walking was impaired. Involuntary movements reduced when lying down in a relaxed posture. Clonazepam was unbeneficial.

### Maternal grandmother (II:2)

This 54-year-old lady was diagnosed with ‘dominant cerebellar ataxia’ aged 43 years. She reported unusual gait of many years’ duration, which had slowly progressed. From infancy, she was described as ‘fidgety’. Symptoms were worse when tired and unrelieved by alcohol. She suffered periodic bouts of significant mood disturbance requiring antidepressants. Her movement disorder worsened considerably in the 12 years since first assessed by a neurologist. Her gait was broad-based and speech dysarthric. There was no nystagmus, but she had interrupted pursuit eye movements. She exhibited titubation, prominent orofacial dyskinesia and intermittent myoclonic jerks of her limbs. She had normal sensation and fundoscopy and no pyramidal tract signs. Genetic testing for spinocerebellar ataxia types 1, 2, 3, 6, 7 and 17 was normal, as was brain MRI. Her sister was said to have similar symptoms but was unavailable for assessment. Her father was said to have had multiple sclerosis, was wheelchair-bound and died of myocardial infarction aged 54. Her mother died aged 47 from an unspecified brain condition, and four maternal aunts were said to have had ‘seizures’.

### Proband’s half-sister (IV:2)

This 5-year-old girl was referred with similar dyskinetic symptoms to her relatives. She had a 1-year history of paroxysmal unsteadiness associated with dystonic foot posturing and dysarthria with unusual facial movements and dribbling, lasting hours in duration. Neurological examination was normal at assessment. However, her reported symptoms clearly suggested she had inherited the same dyskinetic syndrome.

## Materials and methods

### Patients

**Fig. 1:**
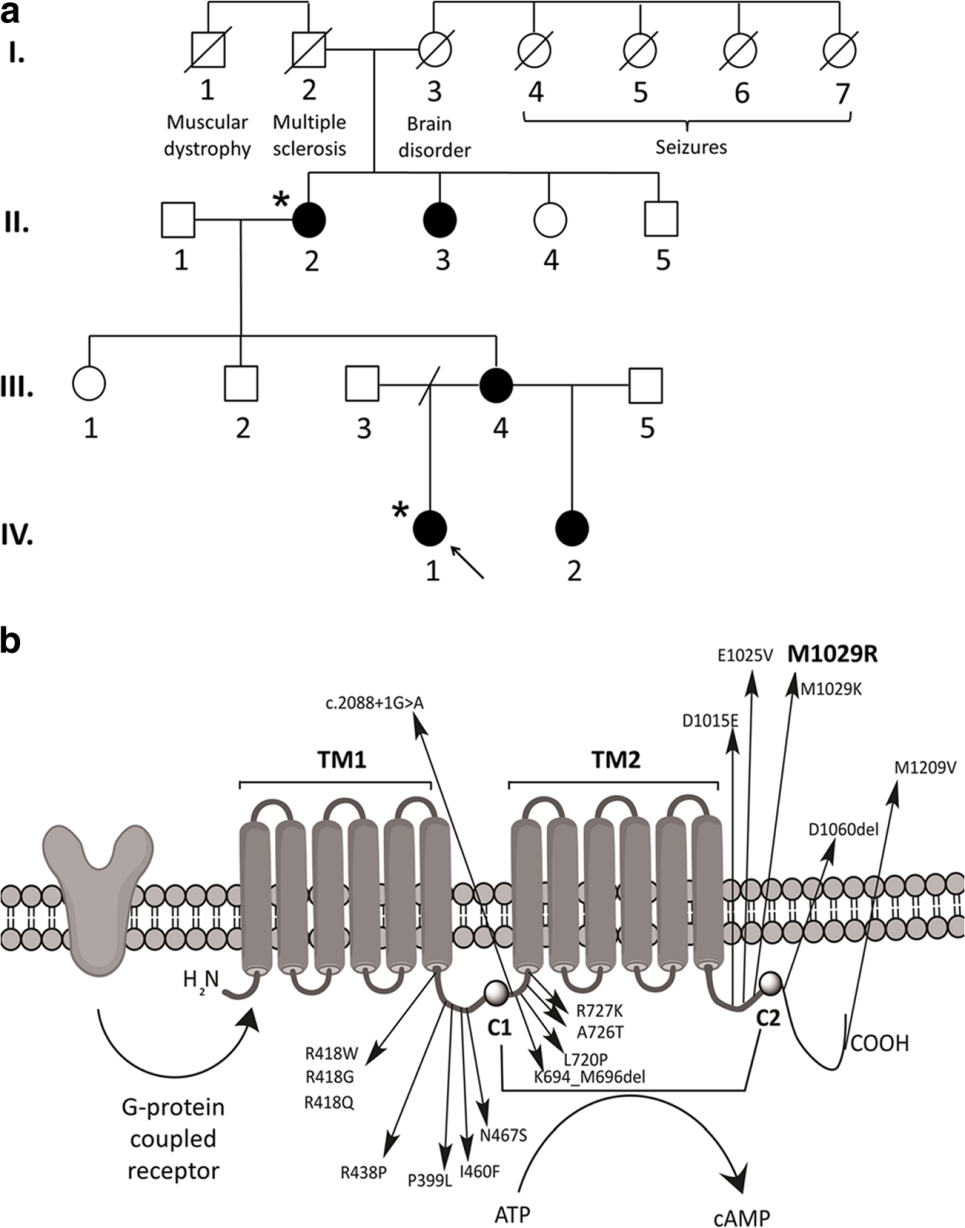
**a** Pedigree of the family described in this case report. Affected individuals are *shaded*, all of whom have had molecular confirmation of the *ADCY5* mutation except for individual II.3, who has not been assessed clinically but is said to have similar symptoms to individual II.2. The precise phenotypes of individuals in generation I are uncertain, relying on familial hear-say, and no medical records were available for review. *Asterisks* indicate individuals that underwent WES (IV.1 and II.2). **b** Adenylyl cyclase (AC5) protein domains and locations of previously reported *ADCY5* disease-causing mutations, including the novel p. M1029R variant observed in this family. The G-protein coupled receptor stimulates AC5 protein activity. AC5 contains two six-helical transmembrane domains (TM1 and TM2) and two cytoplasmic catalytic domains (C1 and C2). The two cytosolic domains form a pocket to convert ATP to cAMP. The *ADCY5* mutation segregating in our family causes a T > G change at nucleotide position 3086 in exon 18, leading to a methionine to arginine change at codon 1029. The variant occurs in the second cytoplasmic loop [2]

Affected individuals (Fig. 1a) were assessed through Wessex Clinical Genetics Service, Wessex Neurological Centre, Southampton Children’s Hospital (all Southampton) and the Nuffield Department of Clinical Neurosciences (Oxford).

### Whole-exome sequencing

Peripheral blood DNA of 20 μg was collected from IV.1 and II.2. WES was performed by Agilent SureSelect Human All Exon 51 Mb V5 capture kit, sequencing with the Illumina HiSeq 2000. Data were processed as previously described [6, 7]. Briefly, paired-end reads were mapped to reference genome hg19 with Novoalign V2.08.02 and variants called with SAMtools 0.1.19. Called SNVs and indels were annotated using ANNOVAR (2013Aug23 version). A local Southampton clinical exome database (*n* = 422) was used to filter regional variation. A tiered analytical approach was employed. Tier 1: analysis of *SGCE*, known to cause myoclonus-dystonia and previously Sanger sequenced. Tier 2: analysis of 21 genes selected from HGMD Professional 2015.3 (BIOBASE Biological Databases) [8], using ‘myoclonus dystonia’ as an analytical search term (Supplemental Table 1). Tier 3: pan-exome extraction and interrogation of all variants shared between proband and grandmother.

## Results

### Tiered WES analysis

*Tier 1.* No *SGCE* coding variants were found*,* consistent with previous Sanger sequencing. Notably, this family’s inheritance pattern included two generations of maternal transmission, making *SGCE* unlikely given its maternal imprinting and almost exclusive paternal expression. *Tier 2.* 18 variants were identified across 10/21 candidate genes. However, all 18 were filtered as unlikely causal based on minor allele frequencies being >0.01 in the 1000 Genomes Project. *Tier 3.* Given the pedigree’s dominant segregation, the causal mutation was expected to be heterozygous, in a well-conserved residue and absent from the local reference exome database (*n* = 422). Eighteen thousand shared variants between the grandmother-granddaughter pair were filtered (Supplemental Fig. 1), giving a final list of 13 (Supplemental Table 3). Comprehensive literature review excluded variants within genes functionally irrelevant to phenotype (Supplemental Table 3). A novel nonsynonymous *ADCY5* variant (c.3086T>G, p. M1029R) was consistently annotated as deleterious by in silico prediction (SIFT, GERP, PolyPhen-2 and MutationTaster). This variant was particularly interesting given *ADCY5*’s known association with FDFM. The mutation was confirmed by Sanger sequencing and heterozygous status demonstrated in all four relatives.

### Treatment with propranolol

Following molecular diagnosis of *ADCY5*-related dyskinesia, the proband was commenced on propranolol 20 mg b.d. with subsequent reduction in all movement-related symptoms. The dose was increased to 30 mg b.d. with sustained effect for 3 months. However, after this time, there was gradual recurrence of symptoms, and unfortunately, these did not respond to increased dosing with 40 mg b.d.

## Discussion

*ADCY5*-related dyskinesia is an autosomal dominant movement disorder where symptoms worsen with anxiety [9]. We describe a three-generation family with this condition presenting primarily as myoclonus-dystonia. In this study, contemporary sequencing technology and unbiased filtering in an affected grandmother-granddaughter pair identified a causative *ADCY5* variant. The T>G substitution causes replacement of hydrophobic methionine with positively charged arginine at a highly conserved residue within the protein’s second cytoplasmic loop (Fig. 1b). No previous evidence of this variant has been found in local or public databases.

Myoclonus-dystonia is a condition characterised by the presence of both myoclonus (rapid muscle contractions of brief duration) and dystonia (sustained and often repetitive twisting movements resulting in abnormal posture) [10]. Abnormal movements tend to affect the upper limbs, trunk and neck, with relative sparing of the lower limbs. Myoclonus-dystonia classically shows a marked improvement of symptoms with alcohol and is also commonly associated with psychiatric comorbidities such as depression, anxiety and obsessive compulsive disorder. Onset is typically in the first two decades of life [11]. Classical myoclonus-dystonia is caused by heterozygous mutations in *SGCE*, and the condition can be inherited in an autosomal dominant fashion but with greatly reduced penetrance in the case of maternal transmission owing to maternal imprinting of *SGCE*. However, both myoclonus and dystonia have also been previously described in cases of *ADCY5*-related dyskinesia (Table 1), suggesting that this gene should also be considered in such cases.

**Table 1:** Confirmed familial and simplex cases of *ADCY5*-related dyskinesia listed by reference and mutation

Study	New cases	No. cases genotyped (affected)	*ADCY5* mutation	Age of onset	Reported involvement (features present in one or more affected individuals within a series)									
					Chorea	Dystonia	Myoclonus	Axial hypotonia	Dysarthria	Face	Head/neck	Gait	Dev. delay	Other features
Chen et al. [1]	1 Family	10 (19)	c.2176G>A p. A726T	2.5–19 years	+	+				+				Dilated cardiomyopathy
Chen et al. [2]	2 Sporadic (1 Mosaic)	2	c.1252C>T p. R418W	19 months to 5 years	+	+	+	+	+	+	+	+	+	Resting tremor Disturbed sleep Camptocormia
Carapito et al. [3]	1 Family	2	c.2088 + 1G>A	6 months to 4 years	+	+				+	+	+		Right leg muscular atrophy Hypotonia (unspecified)
Mencacci et al. [4]	1 Family (1 Mosaic) 1 Sporadic	3	c.1252C>T p. R418W	1–2 years	+	+			+	+		+	+	Ocular impersistence Abnormal saccades Impaired tandem walk
Chen et al. [5]	1 Family (1 Mosaic) 8 Sporadic (3 Mosaic)	16	c.1252C>T p. R418W	6 months to <20 years	+	+	+	+	+	+	+	+	+	Reduced IQ Psychosis
	3 Sporadic (1 Mosaic)	3	c.1253G>A p. R418Q	0.2–1.5 years	+	+	+	+	+	+				Mildly reduced cognition
	1 Sporadic	1	p. R438Pa	2 years		+								Tremulous dystonia
	1 Sporadic	1	p. L720Pa	0.2 years		+	+	+	+					
	1 Family (1 Mosaic)	6 (12)	c.2176G>A p. A726T	1–5 years						+				Essential hereditary chorea family
	1 Family (1 Mosaic)	4	c.3086 T>A p. M1029 K	6 months to 2 years	+	+		+	+	+	+	+	+	Contractures Psychotic depression Reduced MMSE
Chang et al. [12]	2 Families 2 Sporadic	6	c.1252C>T p. R418W	6–14 months	+	+		+	+	+	+	+	+	No unaided walking Abnormal saccades Lower limb spasticity Disturbed sleep Intellectual disability
	1 Family	3	c.1252C>G p. R418G	6 months	+	+	+	+	+	+	+	+		Disturbed sleep Aggression
	1 Sporadic	1	c.1253G>A p. R418Q	6 months	+				+	+	+	+		Disturbed sleep
Dy et al. [13]	1 Sporadic	1	c.2080_2088del p. K694_M696del	5 months	+			+	N/A	+	+	+	+	Significant cognitive delay Severe insomnia Gastrostomy Nonverbal Nonambulatory
	1 Sporadic	1	c.1252G>T p. R418W	1 year	+	+	+	+	+	+	+	+	+	Disturbed sleep Strained/strangled voice
Zech et al. [15]	1 Family	2	c.2180G>A p. R727K	29 years		+					+			Head tremor
	1 Sporadic	1	c.1378A>T p. I460F	4 years		+	+	+	+	+	+	+	+	Restless arms
	1 Sporadic	1	c.1196C>T p. P399L	50 years		+				+	+			
	1 Sporadic	1	c.1400A>G p. N467S	26 years		+					+			
	1 Sporadic	1	c.3177_3182delTGA p. D1060del	58 years		+					+			
	1 Sporadic	1	c.3625A>G p. M1209 V	53 years		+					+			
Meijer et al. [14]	1 Sporadic	1	p. R418W	<1 year	+	+	+	+	+		+	+	+	Impaired vertical eye movements Disturbed sleep
Westen-berger et al. [16]	1 Sporadic	1	c.3045C>A p. D1015E	<2 years	+	+			+	+			+	Alternating hemiplegia of childhood ADHD Hypotonia (unspecified)
	1 Sporadic	1	c.3074A>T p. E1025V	3 months	+	+			+	+			+	Alternating hemiplegia of childhood ADHD Hypotonia (unspecified)
Current paper	1 FAMILY	4 (5)	c.3086 T>G p. M1029R	2–5 years	+	+	+		+	+	+	+		SEE BELOW
	IV:1		c.3086 T>G p. M1029R	2 year	+	+	+		+	+		+		Jerky eye movements
	III:4		c.3086 T>G p. M1029R	2 year	+	+					+			Impaired tandem walk
	II:2		c.3086 T>G p. M1029R	<5 year		+	+		+	+	+	+		Broad-based gait Interrupted gaze pursuit Depression
	IV:2		c.3086 T>G p. M1029R	4 year					+	+		+		

To the best of our knowledge, based on what is stated in each report, these all represent separate cases. Note that only reports documenting *ADCY5* mutations are listed here and where individual cases are mentioned in more than one report, they are listed here only under the original report to document their *ADCY5* mutation. The details of the family from the current report are given in bold type at the bottom, both as a family group and as individual cases. Phenotypes relate to those reported in any affected members of a given family

*+* feature reported, *dev. Delay* developmental delay (including motor delay), *IQ* intelligence quotient, *MMSE* mini-mental state examination, *N/A* not applicable, *ADHD* attention deficit hyperactivity disorder

^a^These variants were listed as being of uncertain significance in Chen et al. [5], although being bioinformatically predicted to be pathogenic

Our proband presented with a childhood-onset jerky movement disorder where both myoclonus and dystonia were prominent features. Her mother displayed similar dystonic movements, while her maternal grandmother displayed a more ataxic phenotype with associated myoclonus. The family’s clinical features were judged to fall within the spectrum of myoclonus-dystonia at the time of investigation, although clearly their two generations of maternal transmission ran contrary to *SGCE* as a likely cause. There are a number of overlapping features between classical myoclonus-dystonia and *ADCY5*-related dyskinesia. These include the presence of both jerky myoclonic movements and dystonia, early onset, a predilection for upper body involvement with relative sparing of the legs and a relatively benign course. There are, however, also a number of notable differences. These include the very frequent involvement of the face and associated dysarthria in *ADCY5* cases, together with more ataxia and disordered eye movements. It has also been reported that disturbed sleep and axial hypotonia are frequently found in *ADCY5* cases [5, 12]. However, neither of these features were apparent in our family.

For further information regarding *ADCY5* and its protein product, please see Supplementary Data. Previous reports are listed in Table 1. Chen et al. [1] described a missense *ADCY5* mutation (c.2176G>A, p. A726T) co-segregating with disease in a large German-American FDFM kindred [1]. The same group reported a further de novo *ADCY5* mutation (c.1252C>T, p. R418W) in two unrelated patients [2]. Carapito et al. [3] reported a patient with early-onset chorea and dystonia who was found to have a de novo splice site *ADCY5* mutation (c.2088+1G>A) [3]. Mencacci et al. [4] found four separate *ADCY5* mutations in a cohort of BHC patients negative for *NKX2-1* mutations [4]. These mutations were c.1252C>T, p. R418W, detected in two unrelated cases; c.2117C>T, p. A706V; and the variant c.1-5G>C in the 5′ untranslated region, which occurred together with a missense variant c.29C>T, p. P10L, although the phase of the two mutations was unknown.

Recently, Chen et al. [5] reported genotype-phenotype correlations in 50 patients with *ADCY5* mutations, including patients with somatic mosaicism [5]. A p. A726T mutation caused a relatively mild phenotype of hand and facial dystonia and chorea. A moderate-severe disorder was caused by p. R418W and p. R418Q mutations involving axial hypotonia, limb hypertonia, intermittent dyskinesias, myoclonus and chorea. Notably, a p. M1029K mutation caused severe dystonia, chorea and myoclonus. This is the same residue as that affected in this report. However, the substitution is for lysine instead of arginine in our family. Interestingly, both lysine and arginine are positively charged, and these substitutions may therefore have similar effects on the properties of the resulting mutant protein. In the family reported by Chen et al., symptoms were unusually severe, with developmental delay, contractures, reduced cognition and psychotic depression. In comparison, none of our affected family have exhibited any of these particularly severe symptoms, although II:2 has a relapsing mood disorder. This, along with the variable features exhibited between individuals in our report, suggests that alterations at residue 1029 may not necessarily lead to the most severe forms of *ADCY5*-related dyskinesia. Instead, it is likely a number of modifying factors such as specific modifier genes that are involved in determining phenotypic severity. Indeed, Chen et al. reported their family’s symptoms lessened with age in at least one case, suggesting the existence of complex regulatory influences.

In the last several months, a number of further reports of *ADCY5* mutations have been published. Chang et al. [12] describe five more patients with the p. R418W mutation and two additional patients with p. R418G and p. R418Q mutations [12]. Notably, these patients all presented with motor milestone delay. Dy et al. [13] report two patients with p. R418W (one of whom was already reported by Chang et al.) and also a severely affected child with a c.2080_2088del (p. K694_M696del) deletion [13]. Interestingly, this severely affected nonverbal and nonambulant patient received no therapeutic benefit from propranolol or from any other drug. However, the patients in this study did appear to benefit from deep-brain stimulation (DBS), as did another p.418W patient reported by Meijer et al. [14]. Zech et al. [15] identified seven early-onset generalised dystonia cases with novel *ADCY5* mutations (c.2180G>A; p. R727K; c.1378A>T, p. I460F; c.1196C>T, p. P399L; c.1400A>G, p. N467S; c.3177_3182delTGA, p. D1060del; c.3625A>G, p. M1209V) [14]. Notably, the authors of this report highlight that the *ADCY5* clinical spectrum may extend to isolated and focal dystonia presentations. Finally, the phenotypic spectrum of *ADCY5* disorders has recently been extended by Westenberger et al. [16], who describe two patients with *ADCY5* mutations (c.3045C>A, p. D1015E; c.3074A>T, p. E1025V) and who display fetures of alternating hemiplegia of childhood [16].

## Conclusion

This report illustrates the importance of considering *ADCY5*-related dyskinesia in cases that fall within the myoclonus-dystonia spectrum, particularly where *SGCE* analysis is normal. It also adds to the growing literature on the phenotype of individuals with *ADCY5* mutations, which appears to cover an increasingly broad range of movement-related features and which varies greatly in terms of phenotypic severity. The treatment of this condition remains challenging, although DBS shows some promise [13, 14]. Although our proband initially appeared to respond to propranolol, this improvement was not sustained and may in retrospect have been partly attributable to a temporary reduction in anxiety relating to the identification of a definitve diagnosis. We note the original paper describing FDFM reported one affected individual who had modest symptomatic benefit using propranolol but that in at least one other report, there was no response to propranolol [9, 13]. Further research will be needed in order to identify the molecular and neurophysiological mechanisms influencing the phenotypic variability of *ADCY5*-related dyskinesia and to determine whether an effective pharmacological agent can be found to treat this condition.

## Electronic supplementary material

ESM 1 (PDF 656 kb)
