# Supplementary material for: ADCY5-related dyskinesia presenting as familial myoclonus-dystonia
Source: Neurogenetics. 2017 Feb 22;18(2):111–7. doi: 10.1007/s10048-017-0510-z (PMC5359383; doi:10.1007/s10048-017-0510-z)
Supplement: Supplementary file 1 — (PDF 656 kb) [file 10048_2017_510_MOESM1_ESM.pdf]

Article title: *ADCY5*-related dyskinesia presenting as familial myoclonus-dystonia

Journal name: Neurogenetics

Authors: Andrew G. L. Douglas*[1,2], Gaia Andreoletti*[1], Kevin Talbot[3], Simon R. Hammans[4], Jaspal Singh[5], Andrea Whitney[5], Sarah Ennis†[1], Nicola C. Foulds†[2]

Affiliations:
1. Academic Unit of Human Development and Health, Faculty of Medicine, University of Southampton, Southampton, UK
2. Wessex Clinical Genetics Service, University Hospital Southampton NHS Foundation Trust, Southampton, UK
3. Nuffield Department of Clinical Neurosciences, University of Oxford, Oxford, UK
4. Wessex Neurological Centre, University Hospital Southampton NHS Foundation Trust, Southampton, UK
5. Paediatric Neurology, University Hospital Southampton NHS Foundation Trust, Southampton, UK

* Joint first authors
† Joint last authors

Corresponding author:
Dr Nicola Foulds, Wessex Clinical Genetic Service, Level G, Mailpoint 627, Princess Anne Hospital, Coxford Road, Southampton SO16 5YA, UK.
E-mail: nicola.foulds@uhs.nhs.uk
Tel: 023 8120 6170

Supplementary Material

ADCY5 information

ADCY5 encodes adenylyl cyclase type V (AC5) protein (Fig. 1b) [1]. *ADCY5* is known to be highly expressed in striatum [2]. Mice deficient in *Adcy5* exhibit a movement disorder (akin to parkinsonism) that worsens with stress [3]. *ADCY5* comprises 21 exons spanning 173.25 kb of locus 3q21.1. The 1261-amino acid AC5 protein catalyses formation of cyclic adenosine monophosphate (cAMP) from adenosine triphosphate (ATP). The N- and C-termini of AC5 lie intracellularly (Fig. 1b). The central portion comprises two intracellular cyclase homology domains (C1 and C2) interspersed by two membrane-spanning domains (TM1 and TM2) each consisting of six helices. AC5 is highly expressed in myocardium and also in the striatum and nucleus accumbens, where it accounts for over 80% of adenylyl cyclase activity [1].

AC5 becomes activated when the C1 and C2 domains are brought together as a heterodimer forming an ATP-binding pocket. This conformational change is regulated by dopaminergic signalling acting on G-proteins, which either promote this interaction if stimulatory or else prevent it if inhibitory by binding to the C1 domain [4]. Previously reported *ADCY5* mutations are believed to be activating, resulting in increased cAMP levels that contribute to the movement disorder. However, the precise mechanisms of myokymia, neurologic dysfunction and other dyskinesias in this condition have not yet been clearly delineated.

Quality control analysis

Summary statistics for each individual are listed in Supplemental Table 2. Exome data were high quality, evidenced by 90% on target coverage achieving a read a read depth of >20. The two samples selected for exome analysis exhibited expected variant sharing for second-degree relatives. Autosomal and X-chromosome heterozygosity were consistent with gender and did not indicate any sample contamination. The application of the SNP tracking panel [5] confirmed sample provenance.

References

1. Matsuoka I, Suzuki Y, Defer N, et al (1997) Differential expression of type I, II, and V adenylyl cyclase gene in the postnatal developing rat brain. J Neurochem 68:498–506.
2. Chen Y-Z, Matsushita MM, Robertson P, et al (2012) Autosomal dominant familial dyskinesia and facial myokymia. Arch Neurol 69:630–635.
3. Iwamoto T, Okumura S, Iwatsubo K, et al (2003) Motor dysfunction in type 5 adenylyl cyclase-null mice. J Biol Chem 278:16936–16940.
4. Tesmer JJ, Sunahara RK, Gilman a G, Sprang SR (1997) Crystal structure of the catalytic domains of adenylyl cyclase in a complex with Gsalpha.GTPgammaS. Science 278:1907–1916.
5. Pengelly RJ, Gibson J, Andreoletti G, et al (2013) A SNP profiling panel for sample tracking in whole-exome sequencing studies. Genome Med 5:89.

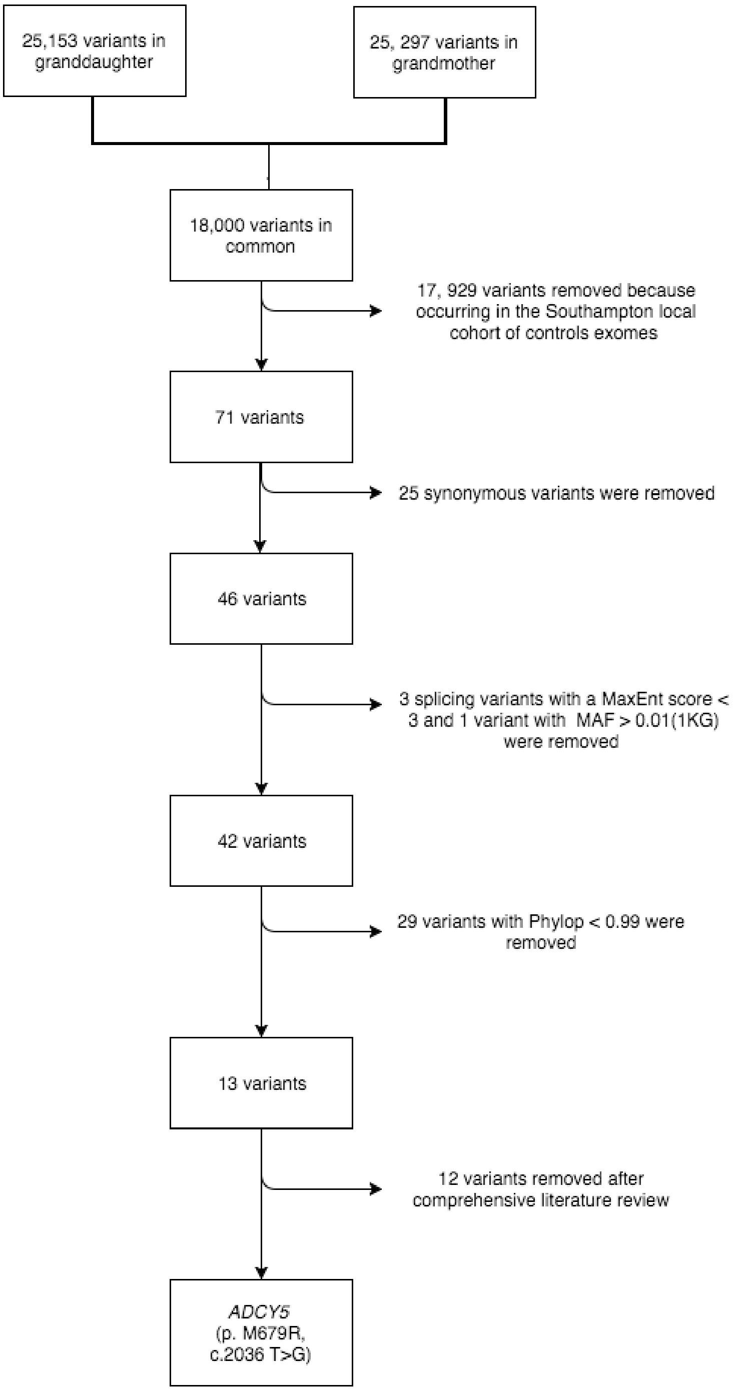

Supplemental Fig. 1 Exome sequencing analysis pipeline. The grandmother-granddaughter pair shared 18,000 variants, of which 17,929 were removed as these were observed in any zygosity state within the local control cohort of exomes (n=422). Of the remaining 71 variants, 25 synonymous variants were discarded due to their low likelihood to impact protein function; 3 splicing variants with a MaxEnt score < 3 were removed and one variant was discounted due to a MAF > 0.01 (1000 Genome Project). Of the 42 variants remaining, 29 were removed due to their low

conservation across species (PhyloP < 0.99). Thirteen variants across 13 genes satisfied the filtering criteria (Table S3). Twelve variants were discounted after literature review as functionally less relevant to the phenotype of this family. The novel nonsynonymous p.M1029R variant in *ADCY5* represented a likely causal variant

Supplemental Table 1 Percentage of coverage for the 21 genes extracted from the HGMD for the search term "myoclonus-dystonia"

Gene symbol	% Coverage Agilent Sure Select All Human Exome V5
**ATP1A3**	100.00
**CACNA1A**	92.11
**EFHC1**	44.82
**GABRA1**	78.02
**GAMT**	53.57
**GCH1**	100.00
**GJD2**	100.00
**GLB1**	96.14
**GNAL**	88.50
**MRE11A**	50.95
**PLA2G6**	95.81
**PRKRA**	100.00
**SCARB2**	97.78
**SCN1A**	98.08
**SGCE**	100.00
**SLC2A1**	83.60
**TH**	100.00
**THAP1**	100.00
**TIMM8A**	90.67
**TOR1A**	100.00
**TUBB4A**	66.55

Supplemental Table 2 Summary statistics for exome sequencing - mapping and coverage. Number of sequenced reads - total number of reads sequenced; Total no. aligned reads - the total number of reads aligned to the reference sequence; Total no. unique align. - the number of reads that uniquely mapped to the reference sequence; Mapped to target reads +/-150bp (%) - the percentage of reads mapped ±150 base pairs to the target; Mapped to target reads (%) - the percentage of reads mapped to the target sequence; Target bases with coverage >1,5,10,20 - the percentage of targets with 1, 5, 10 and 20 read depth; Mean coverage - the mean of the depth coverage

IDs	Number of sequenced reads	Total no. aligned reads	Total no. unique align.	Mapped to target reads (%)	Mapped to target reads +/- 150bp (%)	Mean coverage	Target bases with coverage >1 (%)	Target bases with coverage >5 (%)	Target bases with coverage >10	Target bases with coverage >20	% X Heterozygosity	Pipeline gender	Sample gender	% Autosome heterozygosity	VerifyBamID (freemix)
IV.1	47859430	47609031	46976445	91.99	88.66	66.16	99.43	98.96	98.00	93.46	56.92	F	F	61.36	0.00106
II.2	48106840	47852822	47228795	88.51	91.76	65.93	99.43	98.96	97.99	93.42	61.10	F	F	61.04	0.00273

Supplemental Table 3 Thirteen variants across thirteen genes prioritized after pan-exomic filtering across the two individuals. Logit, PhyloP and GERP are scores for assessing variant deleteriousness; dbSNP137 - rsID in dbSNP 137; Frequency in 1KG - AAF in CEU population in 1000 Genomes Project; Frequency in EVS - AAF in European Americans within the Exome Sequencing Project; Frequency in controls - Southampton non-disease - Frequency in samples without PCA diagnoses. Dots denote missing data. Ns: nonsynonynmous

Gene	Chromosome	Position hg19	Variant type	Coding change	Protein change	Novel (N)	PhyloP	GERP++	dbSNP135	PolyPhen-2	MutationTaster	Frequency in 1KG	Frequency in EVS	Disease associated
ADCY5	3	123010201	ns	c.T3086G	p.M1029R	N	0.997756	4.18	.	0.999	0.99999	.	.	Dyskinesia, familial, with facial myokymia (FDFM)
ELP2	18	33722921	ns	c.A710G	p.E237G	.	0.998524	5.12	rs140841474	0.053	0.012587	0.0005	0.001984	Intellectual disability & eastern equine encephalitis.
FAM45A	10	120877150	ns	c.G452A	p.R151Q	.	0.99439	4.68	rs146864091	0.996	0.9835	0.0005	0.000233	.
HELLS	10	96305697	ns	c.G19T	p.A7S	N	0.999399	2.94	.	0.002	0.000869	.	.	Progressive external ophthalmoplegia, and rothmund-thomson syndrome.
HOXB3	17	46628138	ns	c.G854A	p.S285N	.	0.995955	3.54	rs147425326	0.009	0.067096	.	0.000233	VACTERL association, and myeloid leukemia.
MCRS1	12	49952711	ns	c.A1217G	p.N406S	N	0.997986	4.77	.	0.091	0.999953	.	.	Pigmented basal cell carcinoma, and ampulla of Vater carcinoma
MTSS1L	16	70698029	ns	c.C1795T	p.P599S	N	0.998591	4.24	.	0.999	0.993871	.	.	Metastasis suppressor 1-like
MYO1A	12	57432630	ns	c.G1496A	p.G499D	.	0.998617	3.97	.	0.009	0.772462	.	0.000116	Nonsyndromic hearing loss and deafness
PCDH7	4	30723921	ns	c.A877G	p.I293V	N	0.997756	3.8	.	0.5162	0.013567	.	.	.
SPG20	13	36886588	ns	c.T1510C	p.C504R	N	0.999178	5.7	.	0.927	0.974375	.	.	Troyer syndrome, andspastic paraplegia
SUMF1	3	4403877	ns	c.C1001T	p.S334L	N	0.999786	5.67	.	0.997	0.999922	.	.	Multiple sulfatase deficiency
TMEM117	12	44338025	ns	c.G290A	p.R97Q	N	0.999723	5.47	.	0.389	0.903746	.	.	.
VPS13B	8	100520115	ns	c.T4275G	p.S1425R	N	0.997954	5.29	.	0.6252	0.329101	.	.	Cohen syndrome
